# Extension of the triphasic water potential curve: Accounting for air vapor pressure deficit under soil water stress

**DOI:** 10.1093/plphys/kiaf337

**Published:** 2025-07-31

**Authors:** Steven T Bristow, Thorsten Knipfer

**Affiliations:** Faculty of Land and Food Systems, The University of British Columbia, Vancouver, British Columbia, Canada V6T 1Z4; Faculty of Land and Food Systems, The University of British Columbia, Vancouver, British Columbia, Canada V6T 1Z4

## Abstract

For woody plants subjected to soil dehydration, physiological thresholds of drought-induced stomatal closure (i.e. minimum stomatal conductance, *g*_s-min_) and turgor loss point (TLP) can be derived from the triphasic relationship of stem water potential (Ψ) at midday and predawn, i.e. the “Ψ-curve”. In this study, we provide an extension of the Ψ-curve approach that accounts for vapor pressure deficit (VPD). Experimental data were collected in a greenhouse for potted hazelnut (*Corylus avellana*) trees (varieties “Jefferson” and “Yamhill”)—a species known for its VPD-sensitivity. Consistent with the original Ψ-curve, the “VPD-adjusted” Ψ-curve exhibited a triphasic shape. Predicted thresholds of $\Theta_{1}^{\prime }$ and $\Theta_{2}^{\prime }$ were comparable to independent measures of *g*_s-min_ (−0.86 “Jefferson”; −1.16 MPa “Yamhill”) and TLP (−1.76 MPa “Jefferson”; −2.06 MPa “Yamhill”), respectively. In conclusion, the extended Ψ-curve approach allows the separatation of soil from atmospheric water stress when predicting physiological thresholds using stem water potential.

## Teaser

An extended water potential curve approach that accounts for air vapor pressure deficit when predicting physiological thresholds under drought.

## Introduction

Disentangling atmospheric water demand (i.e. vapor pressure deficit, VPD) from soil water availability when evaluating drought performance of woody crops is important, since rising VPD is as a serious consequence of climate change besides lack of “typical” precipitation events (Novick et al. 2024). Although the emergence of drought stress is closely linked to reductions in soil water availability, drought stress phenomena can be magnified nonlinearly by high VPD conditions (Grossiord et al. 2020; Berauer et al. 2024).

Within the soil–plant–air continuum (SPAC), water inside the plant apoplast (including xylem) is under tension which is predominantly the result of an imbalance between water loss by transpiration and uptake by roots; stored water may be sourced into the transpiration stream to buffer this tension (Holbrook 1995; Kramer and Boyer 1995; Nobel 2009). Transpiration rates depend on VPD and opening/closing of stomata (Nobel 2009). Drought-induced stomatal closure by turgor (*P*) loss of guard cells maximizes leaf resistance to water vapor and minimizes transpirational water loss into a dry atmosphere (Brodribb and Holbrook 2003 ; McDowell et al. 2008 ; Brodribb and McAdam 2011; Buckley 2017; Manandhar et al. 2024). Stomata opening/closing through changes in guard cell *P* are linked to passive (i.e. xylem tension) and/or active (i.e. hormones) signals, and may occur before a decline in drought-induced xylem tension (Passioura 2002). Stomatal closure at a given xylem/apoplast tension is associated with *P* loss of guard cells linked to cellular water loss. Under progressing drought conditions, leaf turgor is eventually lost completely (*P* = 0 MPa; turgor loss point, TLP), which inhibits expansive growth and results in leaf wilting phenomena (Kramer and Boyer 1995; Bartlett et al. 2012). Since xylem tension under drought is affected by both stressors of VPD and soil water availability, it is considered one of the most critical determinants of plant water status. By using a Scholander-type pressure chamber, it has been common practice to estimate xylem tension (i.e. negative pressure) in the main stem of a woody plant by determining “stem water potential” (referred to as Ψ with subscript “PD” or “MD” for predawn or midday, respectively, from here on) as measured on an equilibrated and mature leaf (Scholander et al. 1965; Turner and Long 1980; Kramer and Boyer 1995). Since stomatal regulation and TLP are closely related to Ψ, associated Ψ-thresholds provide fundamental information about a plant's drought response strategies, performance, and survival (Dayer et al. 2020).

**Figure 1.**
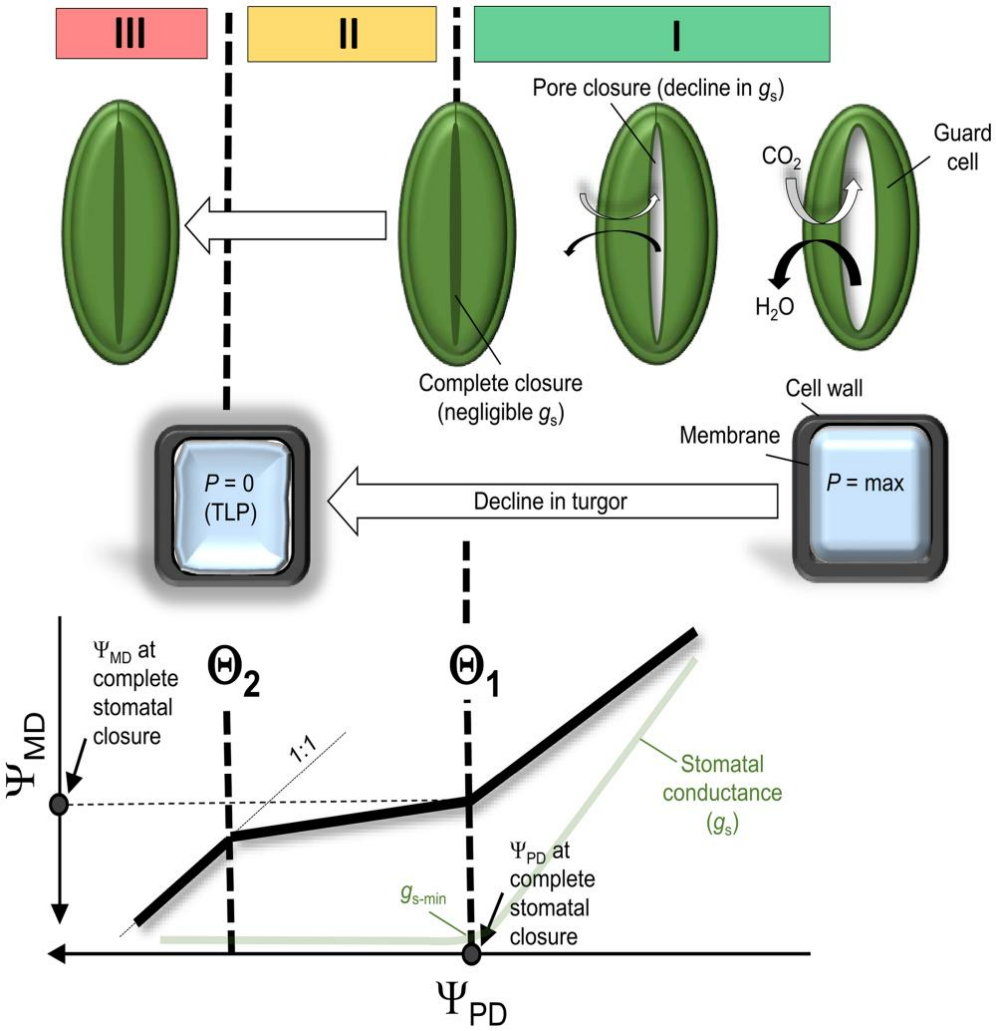
Overview of the water potential (Ψ)-curve approach that predicts “complete stomatal closure” and “turgor loss point” (TLP) under drought. The Ψ-curve originates from the triphasic relationship of stem water potential at midday (Ψ_MD_) and predawn (Ψ_PD_). Thresholds of “complete stomatal closure” at midday and TLP are determined from boundaries (Θ, vertical dashed line) that separate the Ψ-curve into dehydration phases I and II (i.e. Θ_1_) and phases II and III (i.e. Θ_2_) (according to Knipfer et al. (2020)).

There exist several frameworks to characterize drought performance by recognizing a link between Ψ and stomatal regulation (Martínez-Vilalta et al. 2014; Hochberg et al. 2018; Knipfer et al. 2020). Historically, the isohydricity framework has been used to categorize plants into 2 distinct groups (iso- versus anisohydric) according to their ability to regulate Ψ under drought by stomatal responses (Tardieu and Simonneau 1998; Martínez-Vilalta and Garcia-Forner 2017; Hochberg et al. 2018 ). However, Martínez-Vilalta et al. (2014) acknowledged that this strict categorization is unreasonable given the broad spectrum of drought response strategies, and they postulated an alternative approach that quantifies water transport regulation from a linear relationship between Ψ_PD_ and Ψ_MD_ compared with a theoretical 1:1 relationship. For woody plants with a main stem, the triphasic relationship of Ψ_PD_ and Ψ_MD_ (called the “Ψ-curve”) was further explored by Knipfer et al. (2020, 2023). The authors derived Ψ_PD_-thresholds associated with “complete stomatal closure” at midday and TLP by separating this relationship into 3 distinct dehydration phases. We provide an overview of these findings here (Fig. 1):

- Phase I—Ψ_MD_ declines extensively as Ψ_PD_ reaches more negative values. Reductions in midday stomatal conductance (*g*_s_) occur predominantly during phase I. The transition between phases I and II (defined as boundary Θ_1_) coincides with Ψ_PD_ at minimum *g*_s_.
- Phase II—Ψ_MD_ changes are relatively small as Ψ_PD_ becomes more negative. The transition between phases II and III (defined as boundary Θ_2_) coincides with TLP.
- Phase III—Ψ_MD_ and Ψ_PD_ decline in a 1:1 fashion, while *g*_s_ is negligible, and *P* at the leaf level is lost.

Boundaries Θ_1_ and Θ_2_ are obtained using a piecewise linear regression (PLR) model (Knipfer et al. 2020) and are an expression of Ψ_PD_ that provides a surrogate of soil tensions at flow equilibrium (Jones 2007). Charrier (2020) highlighted the usefulness of the Ψ-curve approach for future physiological phenotyping of woody perennials, but raised some concerns about its reliability since (i) effects of VPD on Ψ_MD_ may be substantial when establishing the triphasic curve, resulting in predicted thresholds that are no longer linked to soil water availability and (ii) TLP evaluations should be considered with more caution. In this context, there is an ongoing discussion that false interpretations of indices derived from Ψ are rooted in the failure to separate the drivers of atmospheric (i.e. VPD) and drying soil conditions on Ψ (Hochberg et al. 2018; Mencuccini et al. 2024).

In this study, we provide an extension of the Ψ-curve approach that adjusts Ψ_MD_ for VPD and predicts thresholds of “complete stomatal closure” at midday and TLP associated with soil water stress (see “*Theory*” subsection for details). We tested our extended approach experimentally on hazelnut (*Corylus avellana*) trees, since this woody species is known for its VPD-sensitivity (Pasqualotto et al. 2021; Altieri et al. 2024). Our hypothesis was that VPD impacts the prediction of thresholds in a VPD-sensitive species when using the original Ψ-curve approach by Knipfer et al. (2020). In a greenhouse, potted trees were placed on lysimeters to monitor water loss by evapotranspiration (ET) and soil dehydration (Supplementary Fig. S1 and Table S1). Air temperature (T) and relative humidity (RH) were recorded to determine VPD. Over the experimental period, physiological parameters of Ψ_PD_, Ψ_MD_, and *g*_s_ were measured repeatedly every 1 to 3 d. This was complemented with analyses of leaf sap osmotic pressure (*π*) and TLP from pressure–volume (PV) curves. For trees, VPD-sensitivity was evaluated from the relation of VPD at midday (VPD_MD_) versus Ψ_MD_ (i.e. stem water potential baseline, see Equation (1) for details) and mean daily VPD (VPD_D_) versus normalized evapotranspiration (ET_N_).

### Theory

**Figure 2.**
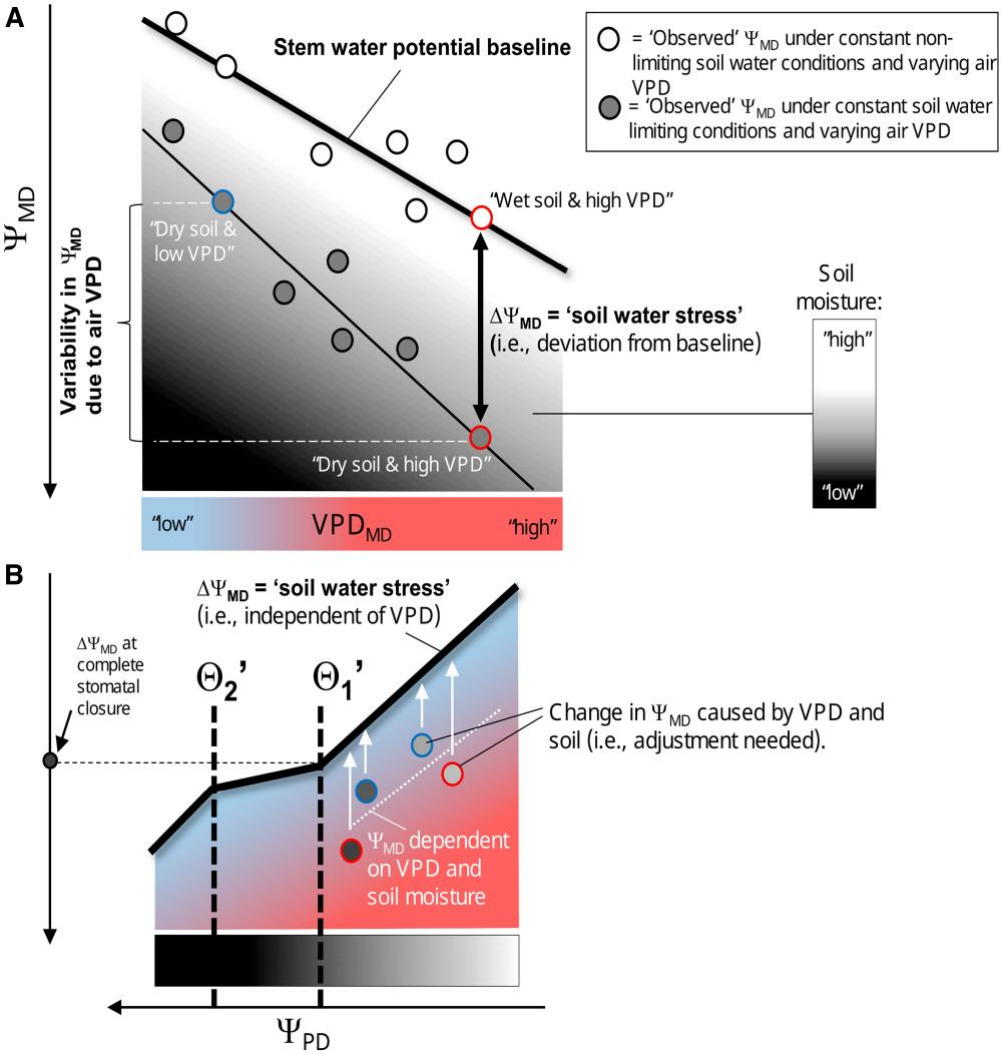
Conceptual scheme of the extended water potential (Ψ)-curve approach. **A)** Variability in Ψ_MD_ (subscript “MD” = midday) in response to VPD and soil moisture. **B)** Extension of the original Ψ-curve (see Fig. 1) that accounts for the contribution of VPD on Ψ_MD_. The extended Ψ-curve is established from the triphasic relationship of ΔΨ_MD_ and Ψ_PD_ (subscript “PD” = predawn) and predicts boundaries $\Theta_{1}^{\prime }$ and $\Theta_{2}^{\prime }$ that are dominated by soil water stress.

Drought conditions are commonly associated with reductions in Ψ_MD_ (i.e. stem xylem tension at midday) caused by a combination of soil and air (in the atmosphere) effects impacting root water absorption (input) and transpiration (output). For that reason, Ψ_MD_ can be variable for a constant soil moisture level under changing VPD_MD_ (Fig. 2). In order to predict physiological responses that are associated with soil water stress, we need to separate air from soil effects on Ψ_MD_ (Fig. 2A).

The contribution of VPD_MD_ to changes in Ψ_MD_ can be accounted for following the “stem water potential baseline” approach (McCutchan and Shackel 1992; Shackel et al. 1997, 2011, 2021). Using a linear regression model, a “stem water potential baseline” (i.e. Ψ_MD_ = *a* + *b* × VPD_MD_) can be established for trees under soil water nonlimiting conditions (i.e. “wet” soil) that experience varying VPD_MD_. Subsequently, a “VPD-adjusted” Ψ_MD_ (i.e. ΔΨ_MD_) can be calculated that represents the soil component of water stress:


(1) $$\Delta \Psi_{\mathrm{MD}}=\Psi_{\mathrm{MD}}-(a+b\times \mathrm{VP}D_{\mathrm{MD}})$$

where *a* is the *y*-intercept when VPD_MD_ is zero, and *b* is the slope value derived from the baseline.

The extended Ψ-curve established from the triphasic relationship of Ψ_PD_ and ΔΨ_MD_ excludes uncertainty due to varying VPD_MD_, and provides for a set of threshold values of “complete stomatal closure” (i.e. $\Theta_{1}^{\prime }$) and TLP (i.e. $\Theta_{2}^{\prime }$) that are exclusive to soil water stress (Fig. 2B). A comparison of Θ_1_ (Fig. 1; Knipfer et al. 2020) with $\Theta_{1}^{\prime }$ (or Θ_2_ with $\Theta_{2}^{\prime }$) provides a means to conclude on the contribution of VPD on these physiological thresholds of the study organism. More details on analyses of Ψ-curves and corresponding boundary values are given in the Material and Methods subsection “*Data analysis*”.

## Results

**Figure 3.**
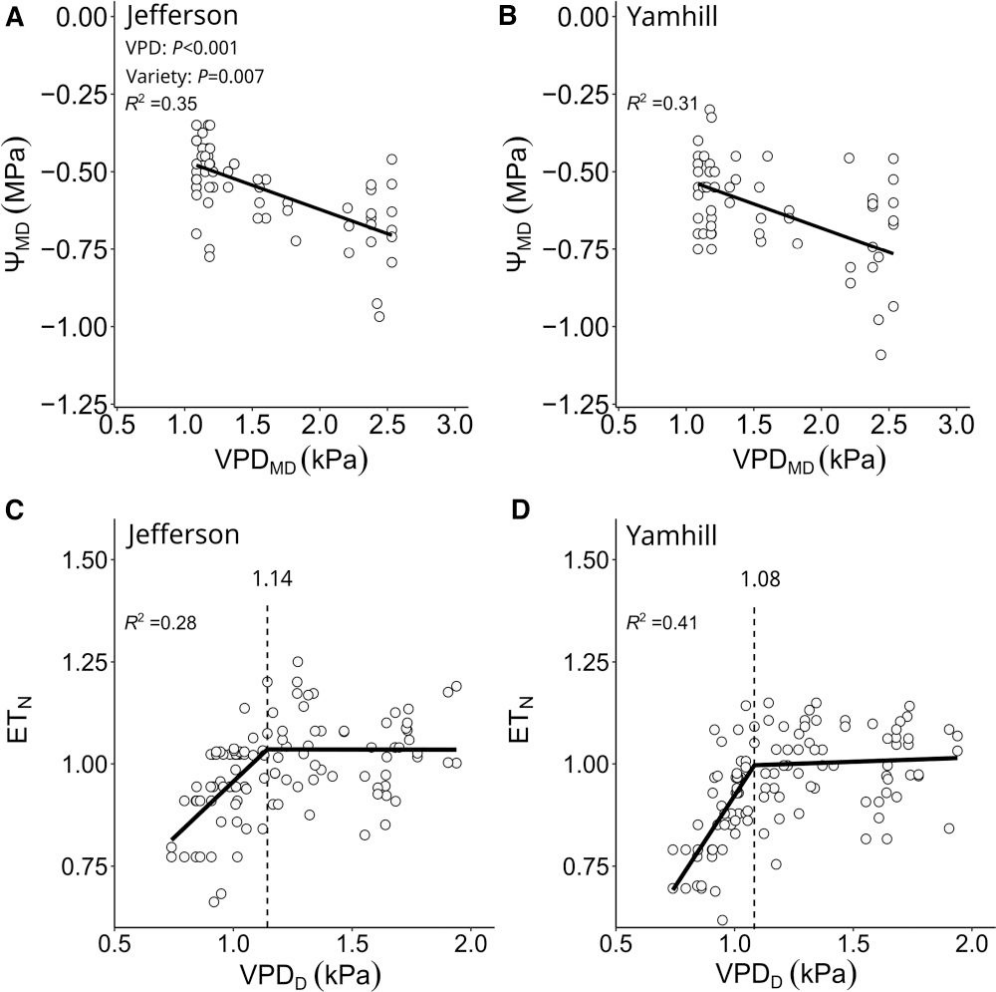
Effect of VPD on stem water potential and evapotranspiration for fully irrigated hazelnut trees of “Jefferson” and “Yamhill”. **A** and **B)** Relationship between midday VPD (subscript “MD”) and midday stem water potential (Ψ_MD_). **C** and **D)** Relationship between daily VPD (subscript “D”) and daily normalized evapotranspiration (ET_N_). Each symbol is an individual measurement. Solid lines are simple **(A** and **B)** and segmented **(C** and **D)** linear regression fitted across data points (*R*^2^ = coefficient of determination). The vertical dashed line is the predicted threshold of ET_N-max_  **(C** and **D)**.

Firstly, we determined the response of Ψ_MD_ and ET_N_ to VPD of hazelnut tree varieties “Jefferson” and “Yamhill” under fully irrigated conditions (Fig. 3). We found that an increase in VPD_MD_ from ∼1 to 2.5 kPa was associated with a decline in Ψ_MD_ by ∼0.2 MPa under fully irrigated conditions (Fig. 3, A and B). For “Jefferson”, the “stem water potential baseline” model predicted a Ψ_MD_ of −0.31 MPa (*a* in Equation (1)) at VPD_MD_ = 0 and a decline of Ψ_MD_ by −0.16 MPa kPa^−1^ with increasing VPD_MD_ (*b* in Equation (1); Fig. 3A). For “Yamhill”, the model predicted a Ψ_MD_ of −0.37 MPa (*a* in Equation (1)) and a decline in Ψ_MD_ by −0.16 MPa kPa^−1^ (*b* in Equation (1); Fig. 3B). On average, Ψ_MD_ was 0.06 MPa higher in “Jefferson compared with “Yamhill” (*P*-value = 0.007). Moreover, an increase in VPD_D_ to ∼1.1 kPa was associated with an increase in ET_N_ in well-watered trees of both varieties; thereafter, increases in ET_N_ with VPD_D_ became negligible (Fig. 3, C and D). Using a PLR model, the critical VPD-threshold was predicted at 1.14 kPa (±0.064 SE; Davies Test *P*-value < 0.001) for “Jefferson” (Fig. 3C) and −1.08 kPa (±0.039 SE; Davies Test *P*-value < 0.001) for “Yamhill” (Fig. 3D). Together, our data indicated that both hazelnut varieties were sensitive to VPD.

**Figure 4.**
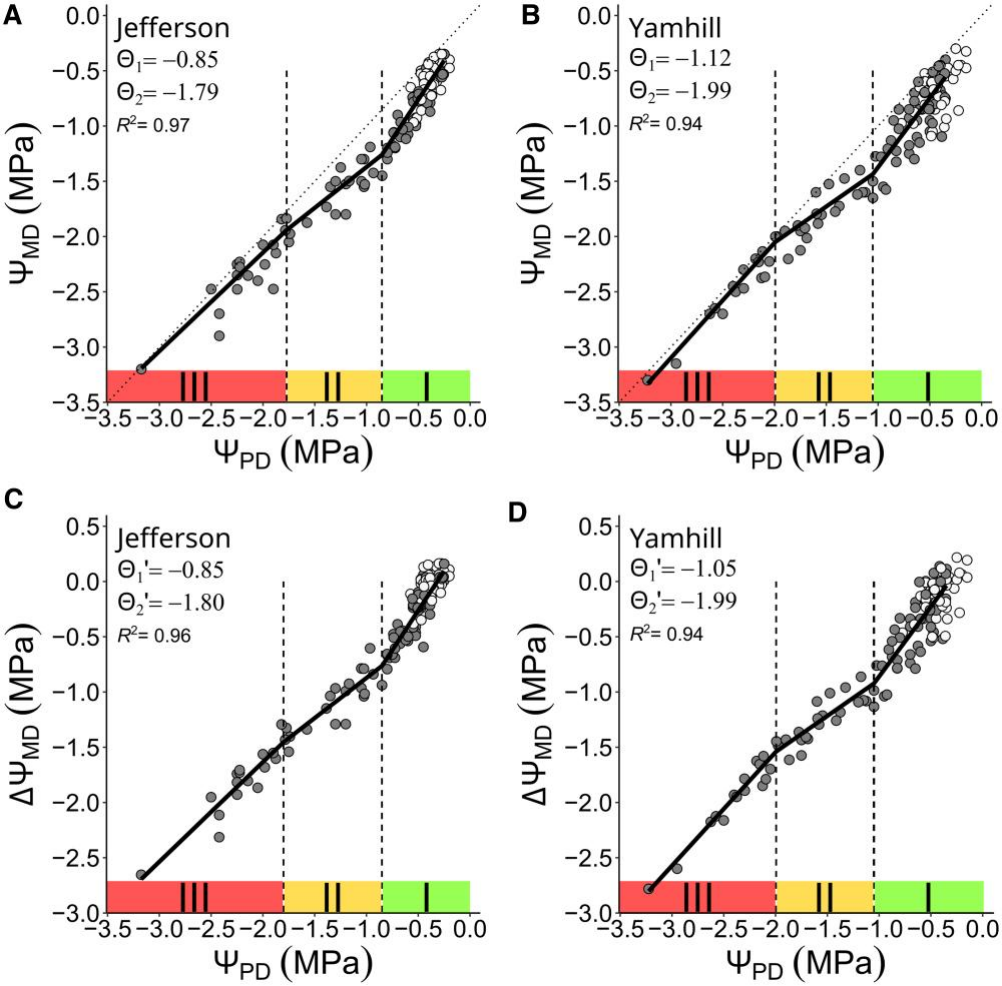
Water potential curves for hazelnut trees of “Jefferson” and “Yamhill”. Curves were generated for measured stem water potential at midday **(**Ψ_MD_, in **A** and **B)** or ΔΨ_MD_ following VPD-adjustment **(**in **C** and **D)**. Each symbol is the measurement of an individual tree at predawn (subscript “PD”) and at midday (white color = fully irrigated trees; gray color = dry-down trees). The solid line is a 3-segmented PLR (*R*^2^ = coefficient of determination). Vertical dashed lines are the predicted boundaries/breakpoints separating the curve into 3 phases indicated by roman numerals.

Secondly, we investigated the relationship of Ψ_PD_ versus Ψ_MD_ (Fig. 4, A and B) and Ψ_PD_ versus ΔΨ_MD_ (Fig. 4, C and D) to confirm that both curves show a triphasic relationship according to a PLR analysis. For “Jefferson”, both Θ_1_ and $\Theta_{1}^{\prime }$ were at −0.85 MPa; Θ_2_ and $\Theta_{2}^{\prime }$ were at −1.79 and −1.80 MPa, respectively (Fig. 4, A and C and Tables 1 and 2). The associated SE for Θ_1_ (0.08) was similar to $\Theta_{1}^{\prime }$ (0.09); same for Θ_2_ (0.32) and $\Theta_{2}^{\prime }$ (0.43). Also, *R*^2^ of the relationship of Ψ_PD_ versus Ψ_MD_ (0.97) and Ψ_PD_ versus ΔΨ_MD_ (0.96) was similar. For “Yamhill”, again, both Θ_1_ and $\Theta_{1}^{\prime }$ were at −1.05 MPa; both Θ_2_ and $\Theta_{2}^{\prime }$ were at −1.99 MPa (Fig. 4, B and D and Tables 1 and 2). The associated SE for Θ_1_ (0.12) was similar to $\Theta_{1}^{\prime }$ (0.15); same for SE of Θ_2_ (0.19) and $\Theta_{2}^{\prime }$ (0.25). The *R*^2^ of the relationship of Ψ_PD_ and Ψ_MD_ (0.96) versus Ψ_PD_ and ΔΨ_MD_ (0.94) was similar.

**Table 1.** Threshold parameters (in MPa ± SE) corresponding to complete stomata closure derived from Ψ-curves (Θ_1_ for measured Ψ_MD_, $\Theta_{1}^{\prime }$ for VPD-adjusted ΔΨ_MD_; Fig. 4), stomatal conductance (*g*_s-min_; Fig. 5) and normalized evapotranspiration (ET_N-min_; Fig. 5)

Parameter	Jefferson	Yamhill	*P*-value
Θ_1_	−0.85 ± 0.08	−1.05 ± 0.13	0.102
$\Theta_{1}^{\prime }$	−0.85 ± 0.09	−1.05 ± 0.15	0.132
*g*_s-min_	−0.86 ± 0.12	−1.16 ± 0.16	0.081
ET_N-min_	−0.90 ± 0.08	−1.22 ± 0.11	0.013

Data are shown for 2 hazelnut varieties, “Jefferson” and “Yamhill”. For each parameter, *P*-values indicate statistical comparison between varieties (see “*Data analysis*” for details).

**Table 2.** Threshold parameters (in MPa ± SE) corresponding to the leaf TLP (in MPa) derived from Ψ-curves (Θ_2_ for measured Ψ_MD_, $\Theta_{2}^{\prime }$ for ΔΨ_MD_; Fig. 4), and compared with the subtraction method (TLP_S_; Fig. 7) and the PV curve method (TLP_PV_; Supplementary Table S2 and Fig. 7)

Method	Jefferson	Yamhill	*P*-value
Θ_2_	−1.80 ± 0.32	−1.99 ± 0.19	0.301
$\Theta_{2}^{\prime }$	−1.80 ± 0.43	−1.99 ± 0.25	0.353
TLP_PV_	−1.76 ± 0.06	−2.06 ± 0.08	0.003
TLP_S_	−1.92 ± 0.09	−2.23 ± 0.10	0.011

Data are shown for 2 hazelnut varieties, “Jefferson” and “Yamhill”. For each parameter, *P*-values indicate statistical comparison between varieties (see “*Data analysis*” and Supplementary Table S2 for details).

**Figure 5.**
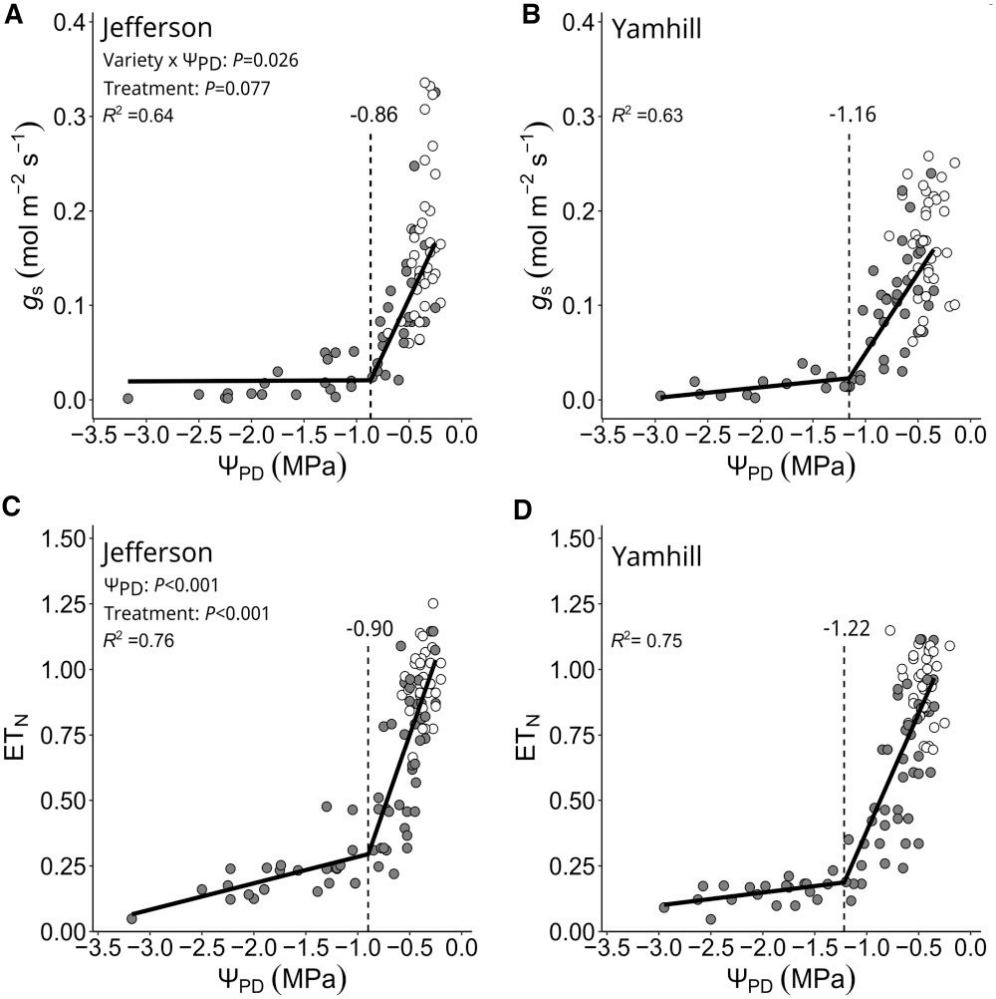
Drought-induced stomatal behavior for hazelnut trees “Jefferson” and “Yamhill”. **A** and **B)** Relationship of stomatal conductance (*g*_s_) and stem water potential at predawn (Ψ_PD_). **C** and **D)** Relationship of normalized evapotranspiration (ET_N_) and Ψ_PD_. Each symbol is the measurement of an individual tree from the same day (white color = fully irrigated trees; gray color = dry-down trees). The solid line is a 2-segmented PLR (*R*^2^ = coefficient of determination), and the vertical dashed line is the predicted breakpoint (*g*_s-min_ in **A**, **B**; ET_N-min_ in **C** and **D)**. For details, see “*Data analysis*”.

**Figure 6.**
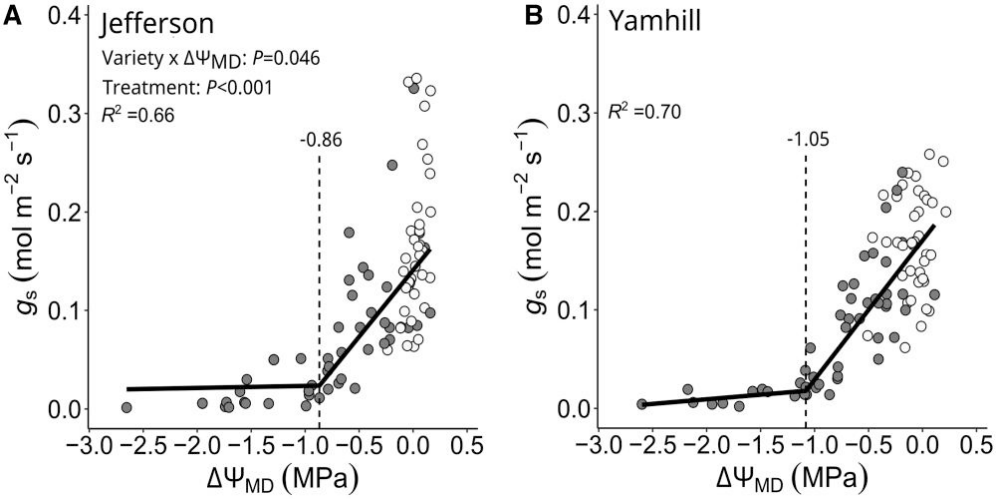
Stomatal conductance (*g*_s_) of hazelnut trees “Jefferson” (in **A**) and “Yamhill” (in **B**) in response to a decline in VPD-adjusted stem water potential at midday (ΔΨ_MD_). Each symbol is the measurement of an individual tree from the same day (white color = fully irrigated trees; gray color = dry-down trees). The solid line is a 2-segmented PLR (*R*^2^ = coefficient of determination), and the vertical dashed line is the predicted breakpoint (*g*_s-min_). For details, see “*Data analysis*”.

Thirdly, we compared boundary $\Theta_{1}^{\prime }$ (=Θ_1_) with independent measures of *g*_s_ to validate their physiological relevance as thresholds of complete stomatal closure (Fig. 5). For “Jefferson”, *g*_s-min_ was reached at −0.86 MPa (Fig. 5A and Table 1) and differed only by 0.01 MPa from $\Theta_{1}^{\prime }$ (*P*-value = 0.458; Fig. 4C). For “Yamhill”, *g*_s-min_ was reached at −1.16 MPa (Fig. 5B and Table 1) and differed by 0.11 MPa from $\Theta_{1}^{\prime }$ (*P*-value *=* 0.3247). It should be noted that *g*_s-min_ was comparable if expressed on the basis of Ψ_PD_ or ΔΨ_MD_ for both varieties (Figs. 5 and 6). Between varieties, *g*_s-min_ was 0.3 MPa greater in “Jefferson” compared with “Yamhill” (*P*-value = 0.088) and following the same trend as observed for $\Theta_{1}^{\prime }$ (Table 1). Moreover, ET_N-min_ was −0.90 MPa for “Jefferson” (Fig. 5C and Table 1; Davies Test *P*-value< 0.001) and 1.22 MPa for “Yamhill” (Fig. 5D and Table 1; Davies Test *P*-value < 0.001) and comparable to *g_s_*_-min_, which further highlights that the contribution of VPD to the threshold determination of complete stomatal closure was negligible (as concluded above from Θ_1_ versus $\Theta_{1}^{\prime }$) for both varieties of this species.

**Figure 7.**
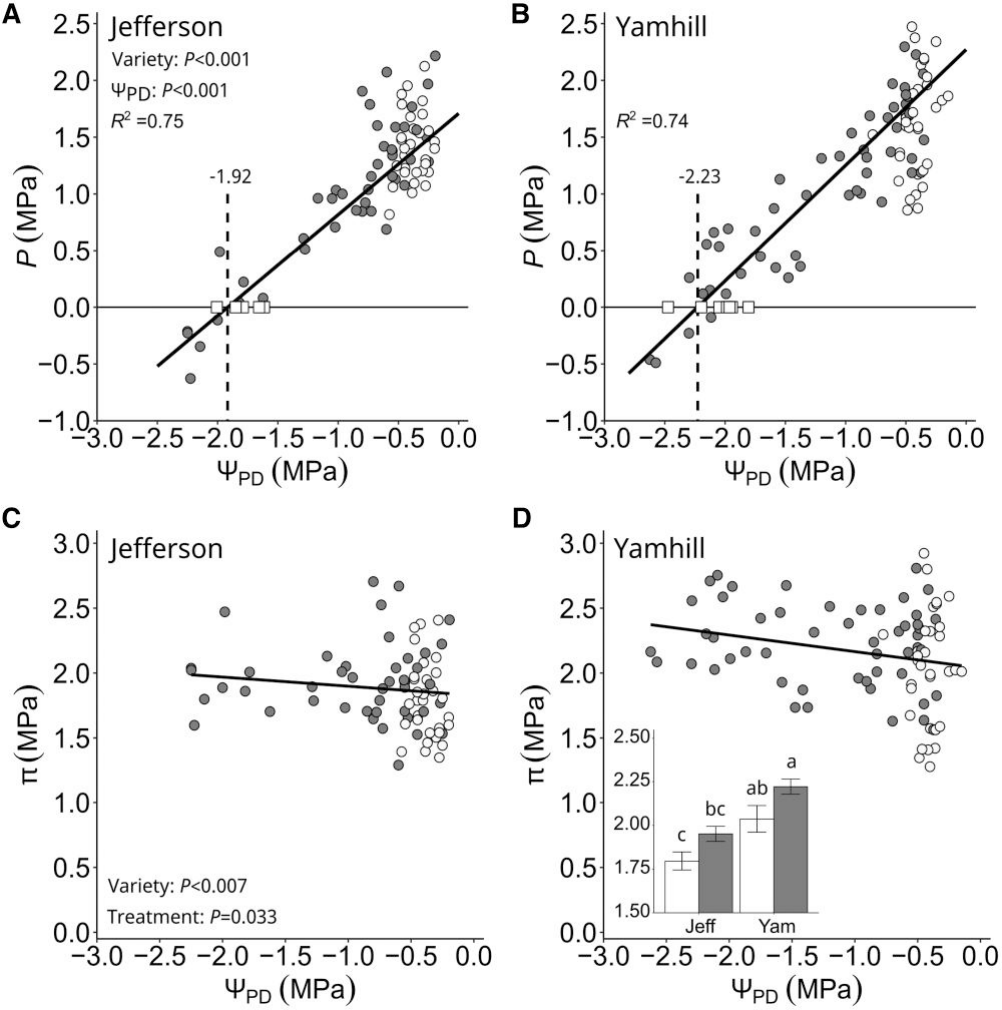
Leaf turgor (*P*) and sap osmotic pressure (*π*) for hazelnut trees “Jefferson” and “Yamhill”. **A** and **B)** Relationship of *P* and stem water potential at predawn (Ψ_PD_). Each circle symbol is the measurement of *P* using the “Subtraction method”. The bold solid line is a simple linear regression (*R*^2^ = coefficient of determination) fitted through all circle symbols. The vertical dashed line is the predicted turgor loss point (TLP_s_). White square symbols are the turgor loss point (TLP_PV_) of an individual tree using the “PV curve method”. **C** and **D)** Relationship of *π* and Ψ_PD_. The inset in panel **D** is mean *π* ± SE (*n* = 8 trees × 6 measurements for dry-down trees and *n* = 8 trees × 3 to 4 measurements for fully irrigated trees) for “Jefferson” (Jeff) and “Yamhill” (Yam), where white bars are fully irrigated and gray bars are dry-down trees. Main effects (i.e. “Ψ_PD_”, “Variety”, and “Treatment”) were tested using a mixed-effects model with Tukey's Honest Significant Difference used for the post hoc comparison with *α* = 0.05. **A** to **D)** Each circle symbol is the measurement of an individual tree (white color = fully irrigated trees; gray color = dry-down trees).

Lastly, we confirmed that independent assessments of TLP using the PV curve and the subtraction method were similar to $\Theta_{2}^{\prime }$ (=Θ_2_) (Fig. 7). Using the “subtraction” method, TLP_s_ were at −1.92 MPa for “Jefferson” (Fig. 7A and Table 2) and −2.23 MPa for “Yamhill” (Fig. 7B and Table 2), and less negative in “Jefferson” (*P*-value = 0.011, Table 2). The difference between $\Theta_{2}^{\prime }$ and TLP_S_ was 0.10 MPa for “Jefferson” (*P*-value = 0.396) and 0.24 MPa for “Yamhill” (*P*-value = 0.195). Using the “PV curve” method, TLP_PV_ was −1.76 MPa for “Jefferson” and −2.06 MPa for “Yamhill”, and again less negative in “Jefferson” (*P*-values = 0.003, Table 2 and Supplementary Table S2). The difference between $\Theta_{2}^{\prime }$ and TLP_PV_ was only 0.04 MPa in “Jefferson” (*P*-value = 0.464) and 0.07 MPa in “Yamhill” (*P*-value = 0.397). For completion, but not necessarily related to the Ψ-curve, we want to report that the relationship between *P* and decreasing Ψ_PD_ showed a linear decline in *P* (Fig. 7, A and B), on the other hand, there was a slight trend that *π* increased consistently with decreasing Ψ_PD_ in both varieties (Fig. 7, C and D). On average, *π* was 0.19 MPa higher in the dry-down compared with the fully irrigated treatment across varieties (*P*-value = 0.042), and *π* of “Jefferson” was consistently ∼0.25 MPa lower than “Yamhill” (*P*-value = 0.012) across both treatments (Fig. 7D, inset).

## Discussion

Accounting for VPD effects on Ψ_MD_, our extension of the original Ψ-curve approach (see Knipfer et al. 2020) provides for drought-induced thresholds of “complete” stomatal closure at midday ($\Theta_{1}^{\prime }$) and TLP ($\Theta_{2}^{\prime }$) in response to soil water stress. Although hazelnut trees were found to be VPD-sensitive (Fig. 3), the comparison of Θ_1_ and $\Theta_{1}^{\prime }$ (same value) suggested that for this species, “complete stomatal closure” in response to a progressive soil dry-down was exclusively linked to soil water stress (Fig. 3 and Table 2); the same can be concluded for TLP (i.e. Θ_2_ and $\Theta_{2}^{\prime }$). At *g*_s-min_, Ψ_PD_ was similar to VPD-adjusted water stress (ΔΨ_MD_; i.e. soil moisture exclusively) (Figs. 5 and 6). When using the Ψ-curve approach, please recognize that this approach does not provide for a threshold of Ψ (or soil water content) at predawn at which stomata “begin” to close (see Fig. 1) (Manandhar et al. 2024). Furthermore, for the successful application of the original and/or extended Ψ-curve approach, we recommend the following:

1. Execute the Ψ-curve approach in response to a progressive dry-down, that does not involve additional irrigation events over the course of the experimental period; if so, this may induce short cycles of recovery events linked to transient root rehydration, stomata opening, and/or a certain degree of recovery in Ψ_MD_ and Ψ_PD_ depending on the intensity of irrigation and time of measurement after the irrigation event.
2. Realize that soil mix and pot size affect the temporal dynamics of the dry-down when planning your dry-down experiment by impacting water availability. Acknowledge that a soil mix of relatively small pore sizes results in a slower dry-down and typically requires higher xylem/apoplast tensions to overcome the stronger matric forces between soil particles for root water extraction, vice versa; even if the exact soil hydraulic properties are unknown. For testing the Ψ-curve approach, we used a combination of soil mix and pot size that facilitated a progressive dry-down (i.e. no irrigation) of around 2 wk, which was necessary to collect a sufficient amount of physiological datapoints and limit pre-experimental water stress. Recent reports suggest that full stomatal closure coincides with a root–soil hydraulic breakdown (Carminati and Javaux 2020; Rodriguez-Dominguez and Brodribb 2020; Javaux and Carminati 2021), which may also be the case for hazelnut, given that Θ_1_ = $\Theta_{1}^{\prime }$.
3. Incorporate sufficient replicate plants to achieve an even distribution of observations in phases I, II, and III and to avoid substantial levels of leaf removal over the experimental period. This can be done by alternating measurements between replicate plants until reaching *g*_s-min_, and subsequently, measure all plants daily as soon as phase II is reached. The duration of plants in phase I is commonly much longer than in phases II and III. Here, it took ∼9 to 10 d to reach Θ_1_ (i.e. at FC of ∼30% for “Jefferson” and 25% for “Yamhill”) and only ∼2 to 4 d to reach Θ_2_ (i.e. at FC of ∼20% of “Jefferson” and ∼18% for “Yamhill”) (Supplementary Fig. S2). The different time periods for dehydration phases is expected as soils exhibit a nonlinear relationship between soil water content and soil matric (water) potential. Furthermore, a decline in *g*_s_ during phase I reduces transpiration rates, which gradually reduces the rate of plant water consumption and soil water depletion. Following *g*_s-min_, soil water depletion is dominated by evaporation when cuticle transpiration is negligible.
4. Exclude the impact of VPD on Ψ_PD_ by covering plant canopies with a dark plastic sheet at night to eliminate light exposure and facilitate nighttime stomatal closure over the course of the experimental trial. Under these conditions, nighttime transpiration is negligible and Ψ_PD_ serves as a surrogate for soil Ψ (Donovan et al. 2003; Jones 2007). By covering tree canopies at night, we found that predawn VPD (range between 0.67 and 0.76 kPa) did not show a relationship with Ψ_PD_ or nighttime ET (i.e. <10% of total daily ET), which would be expected if stomata were in an open state and transpiration would have been substantial. This supports that Ψ_PD_ measurements were insensitive to VPD.

Stomatal closure at high VPD and reduced transpiration under soil water nonlimiting conditions occurs when water supply to the canopy and release of internally stored water (i.e. hydraulic capacitance at stem level too low) is insufficient to match transpirational water loss (Rodriguez-Dominguez et al. 2016; Hu et al. 2023). This water imbalance causes an increase in xylem tension, and a decline of guard cell *P* mainly due to cellular water loss and a subsequent closure of the stomatal pore (Mott and Peak 2013; McAdam and Brodribb 2015); a process that is mechanistically analogous to stomatal closure under limited soil water availability. For woody species, maximum *g*_s_ has been reported for VPD ranging between 1 and 2 kPa (walnut, Rosati et al. 2006; almond, Romero and Botía 2006; grapevine, Soar et al. 2006; olive, Rodriguez-Dominguez et al. 2019). For hazelnut (*C. avellana*), intra-specific variations in stomatal sensitivity to high VPD may exist (Cincera et al. 2019 ). A reduction in *g*_s_ was reported at air VPD > 2.3 kPa when determined over a diurnal cycle (Altieri et al. 2024). Conversely, Pasqualotto et al. (2021) attributed a decline in canopy conductance (as derived from sap flow measurements) to stomatal closure at VPD > 0.6 kPa. Typically, trees store water (primarily in sapwood) at night that is released during the day into the transpiration stream, which can create discrepancies between diurnal patterns of sap flow and actual transpiration (Waring and Running 1978; Čermák et al. 2007; Klein et al. 2016). To adjust for these possible diurnal effects, we expressed ET_N_ on a daily basis. Subsequently, our data indicate that VPD-sensitivity of stomata in “Yamhill” and “Jefferson” (i.e. reduction in ET_N_ at air VPD > 1.1 kPa) does not occur exclusively on a diurnal but also on a daily scale. Moreover, Ψ_MD_ of hazelnut trees in our greenhouse trial was sensitive to VPD, and we identified a significant “stem water potential baseline” for both varieties (see Fig. 2, Equation (1)). Sensitivity of Ψ_MD_ to VPD has been reported for several woody crops under field conditions (walnut, *b* = −0.12 MPa kPa^−1^; prune, *b* = −0.12; olive, *b* = −0.18), and our slope *b* predicted a reduction of Ψ_MD_ by −0.18 MPa for an increase in VPD by 1 kPa) (Fereres and Goldhamer 2003; Rosati et al. 2006; Shackel 2011; Shackel et al. 2021). We speculate that the variability in Ψ_MD_ at a given VPD under soil water nonlimiting conditions (see Figs. 2 and 4; Shackel 2011) is associated with variable rates of transpiration between individuals, which is related to differences in canopy size, with root water absorption being the limiting factor. Together, responses of leaf stomata and Ψ_MD_ to VPD should be recognized when evaluating physiological thresholds under soil water stress, and particularly for woody species that are known to be sensitive to hot/dry atmospheric conditions.

For hazelnut (“Tonda Gentile Romana”) experiencing a decline in soil water availability, Catoni et al. (2017) indicated a minimum in g_s_ at Ψ_PD_ of as low as ∼−2.8 MPa. In comparison, we found that g_s-min_ is reached at Ψ_PD_ (or ΔΨ_MD_) of −0.9 MPa for “Jefferson” and −1.2 MPa for “Yamhill”, suggesting that stomata of both varieties are sensitive to mild soil water deficit when compared Catoni et al. (2017). Moreover, we found that Θ_1_ differed by <0.1 MPa from g_s-min_ and ET_N-min_, which was within the typical measuring error (±0.05 MPa) of Ψ with a Scholander pressure chamber (i.e. related to manual determination of the water meniscus on the cut petiole end). For the future, experimental efforts should also target the rhizosphere and elucidate the soil–root hydraulic continuum at Ψ_PD_ of complete stomatal closure (see paragraph above, recommendation 1).

Accumulation of compatible solutes in response to a decline in soil water availability can reduce the risk of cell dehydration and P loss by raising leaf π (Kramer and Boyer 1995). Traditionally, the PV curve method has been used to determine TLP (i.e. Ψ at P = 0). For hazelnut, differences in TLP_PV_ between varieties seem to exist (Tombesi et al. 2023, reported a range of −2.3 to −2.6 MPa; Cincera et al. 2019, reported “Tonda Romana” = −0.84 MPa, “Tonda Gentile delle Langhe” = −1.2 MPa; Cincera et al. 2019). Our TLP_PV_ was −1.8 MPa in “Jefferson” and −2.1 MPa in “Yamhill”. We speculate that some of the differences in reported TLP_PV_ between studies may be associated with methodological differences in sample preparation/hydration and PV curve interpretation. For that reason, we validated our TLP_PV_ by using an alternative method (“subtraction” method), which gave a TLP_S_ of −1.9 MPa in “Jefferson” and −2.2 MPa in “Yamhill” to further validate our TLP_PV_ (see Fig. 7). Among varieties, “Yamhill” had a more negative TLP compared with “Jefferson” suggesting better drought tolerance. However, a lack of efficient osmo-regulation was evident in both varieties (i.e. only a slight increase in π by on average <0.3 MPa over the entire range of Ψ_PD_, see Fig. 7). Values of TLP_PV_ were within 0.10 MPa of Θ_2_ and $\Theta_{2}^{\prime }$ for both varieties, which highlights that both Θ_2_ and $\Theta_{2}^{\prime }$ are predictors of TLP.

### Future outlook

For a broader application of the Ψ-curve approach, and to overcome some of the current practical obstacles (as listed above), future efforts need to be made to automate recordings of stem water potential for a single individual by employing plant-specific sensors, such as stem psychrometers (Wullschleger et al. 1988 ), micro-tensiometers (Christenson et al. 2024), and/or micro-dendrometers (Gleason et al. 2024). This will also have the advantage of establishing the Ψ-curve by (i) determining Ψ_PD_ and Ψ_MD_ at a given time of the day (as done here) and/or (ii) substituting with daily minimum and maximum Ψ, based on natural diurnal rhythms of plant water status. Either way, this should not be “rushed” because any automated Ψ-curve with its specific curve characteristics will need to be validated with manual measures of Ψ_PD_ and Ψ_MD,_ direct measures of g_s_ and TLP, and may require adjustments to the Ψ-curve approach, such as those presented here. Nevertheless, our long-term efforts to further develop and automate the Ψ-curve approach will broadly benefit physiological phenotyping efforts for drought resistance, and also contribute to closing the “water potential information gap” that currently limits our conceptual understanding of biophysical responses of woody crops within the SPAC (Novick et al. 2022).

## Materials and methods

A list of abbreviations can be found in Table 3.

**Table 3.** List of abbreviations with descriptions used in this study

Abbreviation	Description
Θ_1_	Threshold separating dehydration phases I and II obtained with Ψ_MD_ (MPa)
$\Theta_{1}^{\prime }$	Threshold separating dehydration phases I and II obtained with ΔΨ_MD_ (MPa)
Θ_2_	Threshold separating dehydration phases II and III obtained with Ψ_MD_ (MPa)
$\Theta_{2}^{\prime }$	Threshold separating dehydration phases II and III obtained with ΔΨ_MD_ (MPa)
π	Osmotic pressure (MPa)
Ψ	Water potential (MPa)
Ψ_L_	Leaf water potential determined for the PV curve (MPa)
Ψ_MD_	Stem water potential at midday affected by atmospheric and soil water stress (MPa)
ΔΨ_MD_	Stem water potential at midday dominated by soil water stress (MPa)
Ψ_PD_	Stem water potential at predawn (MPa)
ET	Evapotranspiration (kg d^−1^)
ET_N_	Evapotranspiration to account for transpiration by canopy size (1)
ET_N-max_	VPD-threshold at reduction in evapotranspiration (1)
ET_N-min_	Ψ-threshold at onset of minimum evapotranspiration (1)
FC%	Percentage of soil field capacity (%)
g_s_	Stomatal conductance (mol s^−1^ m^−2^)
g_s-min_	Ψ-threshold at onset of minimum stomatal conductance (MPa)
P	Turgor (MPa)
TLP_PV_	Turgor loss point at leaf level using the PV curve method (MPa)
TLP_S_	Turgor loss point at leaf level using the subtraction method (MPa)
VPD	Vapor pressure deficit (kPa)
VPD_D_	Daily vapor pressure deficit (kPa)
VPD_MD_	Vapor pressure deficit at midday (kPa)

### Plant material and experimental conditions

In March 2022, bare root hazelnut (C. avellana) trees of cultivated varieties “Jefferson” (Mehlenbacher et al. 2011) and “Yamhill” (Mehlenbacher et al. 2009) were transplanted into 7.5-L pots with a growing medium (referred to as “soil” throughout) consisting of a 2:1:1 volume ratio of peat, crushed pumice, and crushed bark, respectively (see Discussion for considerations about soil mix to collect the Ψ-curve); varieties were selected following communications with growers who indicated increased drought sensitivity of “Jefferson” compared with “Yamhill”. In early May 2022, trees were brought into the greenhouse to induce bud break after trees had established outdoors and finished chilling hours. One teaspoon of slow-release fertilizer (4-5-8; Vigoro, the Mosaic Company, Tampa, FL, USA) was added to the soil according to product instructions. Subsequently, a total of n = 31 trees (n = 16 “Jefferson” and n = 15 “Yamhill”) were maintained for 3 months in greenhouse conditions and were watered every 1 to 3 d. In August 2022, experimental trial-1 was initiated over a period of 2 to 3 wk (n = 8 “Jefferson” and n = 7 “Yamhill”). In September 2022, experimental trial-2 was initiated with the remaining trees (n = 8 “Jefferson” and n = 8 “Yamhill” trees). After completion of September 2022 experiments, n = 16 of the 31 trees (n = 8 of each variety) were pruned in October 2022 to main stems, transplanted into 11-L pots, and returned outside until May 2023, where the same procedure as above was followed. The third experimental trial occurred in July 2023 over 2 to 3 wk (greenhouse conditions of temperature, RH, and VPD are summarized in Supplementary Table S1).

Experimental trials-1 to -3 were conducted as follows: Prior to the start of the trial, pots with trees were saturated in the evening, allowed to drain overnight, and then weighed early the next morning to obtain an estimate of total weight at field capacity (m_total-FC_). Trees assigned to the “dry-down” treatment (n = 4 per variety) and half of the trees assigned to the “fully irrigated” treatment (n = 3 to 4 per variety; 2 on lysimeters) were placed on mini-weighing lysimeters to record total pot weight (m_total_) every 30 min and derive percentage of field capacity (FC%, see below) and evapotranspiration over the experimental period. Mini-weighing lysimeters (resolution of 5 g) contained a single arm load cell (Model: YB6-B-35KG-000, Sentran, ON, Canada) connected to an AM16/32 multiplexer controlled by a CR1000 datalogger (Campbell Scientific Inc., Logan, UT, USA), and were each calibrated with known weights of 0, 1, 4.25, and 7.5 kg that covered the range of pot weights. Values of FC% were derived as follows (Sharma et al. 2023):


(2) $$\mathrm{FC}\text{\%}=\;\frac{m_{\mathrm{water}}}{m_{\mathrm{water}\text{-}\mathrm{FC}}}\times 100=\;\frac{m_{\mathrm{total}}-m_{\mathrm{tree}}-m_{\mathrm{plastic}}-m_{\mathrm{dry}}}{m_{\mathrm{water}\text{-}\mathrm{FC}}}\times 100$$

where m_water_ is the soil water weight, m_water-FC_ is the soil water weight at field capacity, m_tree_ is the tree weight (∼100 g), m_plastic_ is the plastic pot weight (80 g), and m_dry_ is the dry soil weight. The water holding capacity of the soil was determined by collecting n = 3 subsamples of ∼300 g from a pot of representative soil compaction that had been saturated and allowed to drain for 48 h, which indicated that m_total-FC_ was composed of 45% of dry soil and 65% of water. Subsequently, m_water-FC_ in Equation (2) was determined by measuring m_total-FC_ times 0.65. For the “dry-down” treatment, water was withheld until leaf senescence began, which was commonly observed around 14 to 20 d in 2022 and 10 to 14 d in 2023. For the “fully irrigated” treatment, pots were watered by hand every 1 to 3 d to keep average daily FC% > 80. A typical time course of daily changes in FC% over trials-1 and -2 is shown in Supplementary Fig. S2.

### Evapotranspiration

Daily ET was normalized to adjust for differences in transpiration associated with canopy size differences between trees (ET_N_, Equation (3)) (Koehler et al. 2023):


(3) $$ET_{N}=\;\frac{ET_{D}}{ET_{i3}}$$

where ET_N_ is normalized daily evapotranspiration, ET_D_ is daily evapotranspiration determined by calculating the difference in m_total_ (as derived from mini-weighing lysimeters) between 12:00 AM and 11:30 PM, ET_i3_ is average ET for the first initial 3 d of the experiment. The first 3 d were used to determine ET_i3_ because ET_D_ was commonly higher on day-1 of watering and reached FC% = 80 (target for control treatment) by ∼day-3 (see Supplementary Fig. S2). The response and corresponding VPD-threshold of ET_N_ to VPD was evaluated from the relationship of ET_N_ and daily VPD (VPD_D_ is the average VPD from 15-min recordings of temperature and RH between 12:00 AM and 11:30 PM) for fully irrigated trees using a PLR model; the Ψ_PD_-threshold at the onset of minimum ET_N_ was derived from the boundary predicted from a 2-segmented PLR model fitted to the relationship of Ψ_PD_ and ET (see “Data analysis”).

### Stem water potential

A Scholander pressure chamber (1505D, PMS Industries, Albany, OR, USA) was used to measure stem water potential (Ψ) at predawn and midday (Fulton et al. 2014; Knipfer et al. 2020, 2023 ). The evening prior to measurements (between 1900 and 2100 h), 2 adjacent mature leaves per tree near the main stem were covered with mylar bags (i.e. aluminum covered with plastic inside) to reduce transpirational water loss and allow for leaves to equilibrate with the stem xylem. Subsequently, tree canopies were covered with a large polyethylene plastic sheet to eliminate light exposure that may trigger stomatal opening, maintain “low” VPD conditions, and consequently minimize nighttime canopy transpiration that would increase stem xylem tension in addition to the soil prior to measurement of Ψ_PD_ (Donovan et al. 2003; Knipfer et al. 2023). This procedure allows for the interpretation of Ψ_PD_ as a surrogate of soil water potential (Jones 2007), and excludes a contribution of predawn VPD on Ψ_PD_ assuming nontranspiring conditions. Bagged leaves were excised from the main stem at predawn (between 5:30 AM and 6:30 AM) to determine Ψ_PD_ and at midday (between 12:00 PM and 1:00 PM) to determine Ψ_MD_. After excision, leaves were sealed into their mylar bags and placed into a cooler with ice packs and transported to the lab, where all leaves were measured within 1 h. For measurement, a leaf was removed from its bag (i.e. to access the relatively short petiole of ∼5 mm), the petiole was quickly recut with a fresh razorblade, and 2 cuts of around 1 cm in length were made on both sides near the central midrib so that the petiole could protrude through the pressure chamber gasket; ideally, the leaf is replaced in its bag prior inserting it into the chamber to eliminate any water loss but this was not done here to avoid twisting the leaf while sealing the chamber lid—in turn, we assumed that water loss from the leaf surface was negligible until measurement and did not impact our Ψ readings. Subsequently, the pressure in the chamber was raised slowly at a constant rate (0.05 to 0.1 MPa s^−1^), and the negative number of the balancing pressure (i.e. Ψ) was recorded when a water meniscus started to form on the cut petiole surface. To avoid excess defoliation, measurements were conducted initially every 2 to 3 d on a selection of n = 2 to 3 trees per treatment combination until soil dryness was suspected to reduce Ψ, at which point all water stress trees were measured every 2 d until leaf senescence.

For each variety, the “stem water potential baseline” was determined for fully irrigated trees (control treatment) using a linear regression analysis, and ΔΨ_MD_ was determined according to Equation (1) (see “Theory” section). Midday vapor pressure deficit (VPD_MD_) was determined based on the average of 15-min temperature and RH readings between 11:30 AM and 1:00 PM. Details on analyses of Ψ-curves and corresponding boundary values are given in the section “Data analysis”.

### Stomatal conductance

A porometer (LI-600, Li-Cor, Inc., Lincoln, NE, USA) was used to determine leaf stomatal conductance at midday (g_s_). For each tree, 1 mature and healthy leaf was selected and carefully marked with a string at the petiole. The selected leaf was measured between 12:00 PM and 1:00 PM, just before Ψ_MD_ measurements. The same leaf was measured over the entire experimental trial and occasionally compared to g_s_ of 2 to 3 similar leaves on the same tree to validate that readings were representative of the canopy. If so, multiple g_s_ measurements on the same day were averaged; if not, the marked leaf was discarded, and a new leaf was selected for subsequent g_s_ measurements. When g_s_ values reached near zero and/or began to produce marginally negative values, that tree was no longer measured for g_s_ (values of g_s_ < 0 were removed for analysis, see “Data analysis”). The Ψ_PD_-threshold at the onset of minimum g_s_ (g_s-min_) was derived from the boundary predicted from a 2-segmented PLR model fitted to the relationship of Ψ_PD_ and g_s_ (see “Data analysis”); we refer to Ψ_gs-min_ also as the threshold of stomatal closure.

### Leaf turgor and osmotic pressure

Every second day of Ψ-measurements, the leaf used for Ψ_PD_ was also used for leaf sap osmotic pressure (π) and leaf turgor (P) using the “subtraction” method (P = Ψ_PD_ − π; Jones and Turner 1978). Immediately after Ψ_PD_ measurement, about half of the leaf lamina was separated from the central midrib (to minimize the contribution of xylem sap), rolled, inserted into a cut pipette tip located in a 2-mL Eppendorf tube, sealed, and placed in the freezer (−20 °C) for 4 to 12 wk. Samples of the leaf lamina were thawed at room temperature for 15 min and then centrifuged (Eppendorf Centrifuge 5417C) at 11,000 rpm for 5 min to extract leaf sap. A volume of 10 µL of extracted sap was placed in an osmometer (Vapro 5600, ELITechGroup Biomedical Systems, Logan, UT, USA) and osmolality was recorded. Osmolality was converted to π according to 407.5 mOsmol kg^−1^ = 1 MPa (Barrios-Masias et al. 2015). Following this procedure for the “subtraction” method, it was assumed that π of extracted leaf sap was representative of symplastic sap at predawn and that Ψ_PD_ measured on an equilibrated nontranspiring leaf was representative of cell Ψ at predawn (Jones and Turner 1978; Knipfer et al. 2020; Sharma et al. 2023). The Ψ_PD_-threshold at P = 0 (TLP_S_) was derived from a linear regression model applied to the relationship of P and Ψ_PD_ (see “Data analysis”).

In addition, the “PV curve method” was used to determine turgor loss point (TLP_PV_) based on 1 to 2 leaves from (n = 4) trees per cultivar on 2 different dates prior to the start of trial-1 (Bartlett et al. 2012). Since hydration of excised leaves can result in artificial filling of intercellular spaces (Arndt et al. 2015), we hydrated 1 leaf from different trees (n = 3 per cultivar) overnight on June 21st by bagging each leaf (as described for Ψ measurements) and watering the tree above FC. The next morning (around 8:00 AM), leaves were cut with a razor blade, placed in a cooler with ice packs, and taken to the lab. Leaf area was measured using a leaf area meter (LI-3000, Li-Cor, Inc., Lincoln, NE, USA). Subsequently, the leaf was placed in a pre-weighed whirl pack bag for 10 min for equilibration, and leaf water potential of the bagged leaf (Ψ_L_) was measured as described above. Immediately after, the leaf inside the bag was weighed with an analytical balance (SI-114, 0.1 mg resolution, Denver Instruments, NY, USA). Then, the leaf was removed from the bag and placed on the lab bench to allow for water loss by transpiration; initially, we kept the time outside the bag brief (∼2 min to capture >0.2 MPa changes in Ψ_L_), but it was gradually increased and exceeded 15 min as Ψ_L_ approached values of −1.5 MPa. This entire process was repeated until Ψ_L_ reached −3.0 MPa. After completion, leaves were placed in an oven at 100 °C for 48 h to determine leaf dry weight. The process was repeated again the following week on (n = 4) trees per cultivar, with leaves from the same trees plus 1 extra. The PV curve was analyzed following Sack et al. (2018) and references therein: The relationship between leaf water weight and Ψ_L_ was determined via linear regression, where the y-intercept of the regression indicates the leaf at full hydration. The weight at full hydration was used to calculate the leaf relative water content (water weight at any time divided by weight at full hydration times 100). The relationship between $\Psi_{L}-1$ and the deviation from 100% relative water content was found by plotting the 2 variables. Subsequently, Ψ_TLP_ was visually determined as the point where the relationship between the 2 variables reached an inflexion point (R^2^ of regression decreased with additional points added to the model). After the inflexion point, greater reductions in relative water content no longer decreased Ψ^−1^ as would be expected when leaf turgor has been lost. A representative PV curve is shown in Supplementary Fig. S3, and a summary of measured PV curve parameters can be found in Supplementary Table S2.

### Data analysis

The effects of treatment by “dry-down”, “Ψ_PD_”, and/or “variety” and their interactions on the response variables π, P, g_s_, Ψ_MD_, and ET_N_ (fixed effects) were analyzed using linear mixed-effects (“lme4” package) and/or PLR (“segment” package) models in R Statistical Software (V4.3.1; R Core Team, 2023, Vienna, Austria). The model type depended on whether the data exhibited a constant linear or nonconstant-linear pattern. PLR models were first conducted as simple linear models and then tested using “Davies.test” (“segment” package) where P-values < 0.05 suggest a possible change in slope (i.e. breakpoint/boundary) but not the number of breakpoints (Muggeo 2008). Simple linear and PLR models were compared using Akaike information criterion for small sample sizes (“AICc” function; “MuMIn” package) and “anova” (base R) functions. The PLR model was selected if the AICc was 1 unit less than the AICc for the simple linear model (Knipfer et al. 2023). Mixed-effects models were treated as “repeated-measures” analysis and random effects were selected using AICc with “experimental trial”, “block”, “pot” (as subject), and/or “day” (as within-subject) factors. In PLR models, possible random effects were treated as categorical covariates and averaged over when plotting the regression line; a regression line was not plotted for fully irrigated plants (note: breakpoints are not determined per categorical variable). When necessary, post hoc comparisons were conducted using the “emmeans” and “multcomp” packages with Tukey's test and α = 0.05.

Varietal comparison of the same breakpoint (e.g. Θ_1_, Θ_2_, g_s-min_, or ET_N-min_) between 2 PLR models was conducted using a test for the equality of regression coefficients similar to Paternoster et al. (1998). The test statistic was calculated using the formula:


(4) $$t=\;\frac{b_{1}-b_{2}}{\sqrt{\;\mathrm{SE}b_{1}^{2}\;+\mathrm{SE}b_{2}^{2}}}\;$$

where t is the test statistic, b is the model parameter (e.g. breakpoint, intercept, or slope), SEb is the standard error of the model parameter. The standard error (SE) for PLR breakpoints was extracted from the variance–covariance matrix of the model, which used the delta method (Muggeo 2008). P-values were determined using the “pt” function (“stats” package) with the degrees of freedom equal to the number of “dry-down” plants minus the number of compared parameters (i.e. degrees of freedom = 24 plants − 2 breakpoints = 22; when comparing within a variety (e.g. Θ_1_ to Θ_2_) degrees of freedom = 12 − 2 = 10). Our approach to the selection of degrees of freedom allows for consistency when comparing breakpoints between varieties as well as within a variety and considers the need to balance between Type 1 and Type 2 error rates.

For TLP_s_ (simple regression model), SE was calculated by bootstrapping model residuals with replacement of 10,000 observations per iteration and 10,000 iterations (“boot” function; “boot” package) to generate a distribution of possible x-intercepts where the 0.025 to 0.975 quantiles are believed to represent a 95% confidence interval for the TLP (Canty 2002). The SE was then calculated using the formula: SE = (CI 97.5% − CI 2.5%)/(1.96 · 2), where 1.96 is the critical value for a Z-distribution with α = 0.05. For TLP_PV_, the SE of the mean for each variety was found as the standard deviation (SD) divided by the square root of the number of leaf samples. Two sample t-tests were then used to compare these thresholds for differences between varieties for means and SE calculated using the same approach.

## Supplementary Material

kiaf337_Supplementary_Data

## Data Availability

The data underlying this article are available in the article and in its online supplementary material.

## Supplementary data

The following materials are available in the online version of this article.

**Supplementary Table S1.** Overview of environmental greenhouse conditions.

**Supplementary Table S2.** Parameters obtained from PV curve analyses of hazelnut leaves.

**Supplementary Figure S1.** Representative image of hazelnut trees placed on mini-weighing lysimeters.

**Supplementary Figure S2.** Representative time course of changes in percentage of field capacity.

**Supplementary Figure S3.** Representative PV curve to derive TLP_PV_.

## Dive Curated Terms

The following phenotypic, genotypic, and functional terms are of significance to the work described in this paper:

stem AmiGo: PO:0009047

stomatal conductance Planteome: TO:0000522

transpiration rate Planteome: TO:0001018

water CHEBI: CHEBI:15377

## References

[kiaf337-B1] Altieri G, Wiman NG, Santoro F, Amato M, Celano G. Assessment of leaf water potential and stomatal conductance as early signs of stress in young hazelnut tree in Willamette valley. Sci Hortic. 2024:327.

[kiaf337-B2] Arndt SK, Irawan A, Sanders GJ. Apoplastic water fraction and rehydration techniques introduce significant errors in measurements of relative water content and osmotic potential in plant leaves. Physiol Plant. 2015:155(4):355–368. 10.1111/ppl.12380 26331213

[kiaf337-B3] Barrios-Masias FH, Knipfer T, McElrone AJ. Differential responses of grapevine rootstocks to water stress are associated with adjustments in fine root hydraulic physiology and suberization. J Exp Bot. 2015:66(19):6069–6078. 10.1093/jxb/erv324 26160580 PMC4615816

[kiaf337-B4] Bartlett MK, Scoffoni C, Sack L. The determinants of leaf turgor loss point and prediction of drought tolerance of species and biomes: a global meta-analysis. Ecol Lett. 2012:15(5):393–405. 10.1111/j.1461-0248.2012.01751.x 22435987

[kiaf337-B5] Berauer BJ, Steppuhn A, Schweiger AH. The multidimensionality of plant drought stress: the relative importance of edaphic and atmospheric drought. Plant Cell Environ. 2024:47(9):3528–3540. 10.1111/pce.15012 38940730

[kiaf337-B6] Brodribb TJ, Holbrook NM. Stomatal closure during leaf dehydration, correlation with other leaf physiological traits. Plant Physiol. 2003:132(4):2166–2173. 10.1104/pp.103.023879 12913171 PMC181300

[kiaf337-B7] Brodribb TJ, McAdam SA. Passive origins of stomatal control in vascular plants. Science. 2011:331(6017):582–585. 10.1126/science.1197985 21163966

[kiaf337-B8] Buckley TN . Modeling stomatal conductance. Plant Physiol. 2017:174(2):572–582. 10.1104/pp.16.01772 28062836 PMC5462010

[kiaf337-B9] Canty AJ . Resampling methods in R: the boot package. R News. 2002:2(3):2–7. https://journal.r-project.org/articles/RN-2002-017/

[kiaf337-B10] Carminati A, Javaux M. Soil rather than xylem vulnerability controls stomatal response to drought. Trends Plant Sci. 2020:25(9):868–880. 10.1016/j.tplants.2020.04.003 32376085

[kiaf337-B11] Catoni R, Gratani L, Bracco F, Granata MU. How water supply during leaf development drives water stress response in Corylus avellana saplings. Sci Hortic. 2017:214:122–132. 10.1016/j.scienta.2016.11.022

[kiaf337-B12] Čermák J, Ku”era J, Bauerle WL, Phillips N, Hinckley TM. Tree water storage and its diurnal dynamics related to sap flow and changes in stem volume in old-growth Douglas-fir trees. Tree Physiol. 2007:27(2):181–198. 10.1093/treephys/27.2.181 17241961

[kiaf337-B13] Charrier G . Extrapolating physiological response to drought through step-by-step analysis of water potential. Plant Physiol. 2020:184(2):560–561. 10.1104/pp.20.01110 33020321 PMC7536708

[kiaf337-B14] Christenson CG, Gohardoust MR, Calleja S, Thorp KR, Tuller M, Pauli D. Monitoring cotton water status with microtensiometers. Irrig Sci. 2024:42(5):995–1011.

[kiaf337-B15] Cincera I, Frioni T, Ughini V, Poni S, Farinelli D, Tombesi S. Intra-specific variability of stomatal sensitivity to vapour pressure deficit in Corylus avellana L.: a candidate factor influencing different adaptability to different climates? J Plant Physiol. 2019:232:241–247. 10.1016/j.jplph.2018.11.019 30544052

[kiaf337-B16] Dayer S, Herrera JC, Dai Z, Burlett R, Lamarque LJ, Delzon S, Bortolami G, Cochard H, Gambetta GA. The sequence and thresholds of leaf hydraulic traits underlying grapevine varietal differences in drought tolerance. J Exp Bot. 2020:71(14):4333–4344. 10.1093/jxb/eraa186 32279077 PMC7337184

[kiaf337-B17] Donovan LA, Richards JH, Linton MJ. Magnitude and mechanisms of disequilibrium between predawn plant and soil water potentials. Ecology. 2003:84(2):463–470. 10.1890/0012-9658(2003)084[0463:MAMODB]2.0.CO;2

[kiaf337-B18] Fereres E, Goldhamer D. Suitability of stem diameter variations and water potential as indicators for irrigation scheduling of almond trees. J Hortic Sci Biotechnol. 2003:78(2):139–144. 10.1080/14620316.2003.11511596

[kiaf337-B19] Fulton A, Grant J, Buchner R, Connell J. Using the pressure chamber for irrigation management in walnut, almond and prune. 2014. 10.3733/ucanr.8503

[kiaf337-B20] Gleason SM, Stewart JJ, Allen B, Polutchko SK, McMahon J, Spitzer D, Barnard DM. Development and application of an inexpensive open-source dendrometer for detecting xylem water potential and radial stem growth at high spatial and temporal resolution. AoB Plants. 2024:16(2):plae009. 10.1093/aobpla/plae009 38510929 PMC10953470

[kiaf337-B21] Grossiord C, Buckley TN, Cernusak LA, Novick KA, Poulter B, Siegwolf RT, Sperry JS, McDowell NG. Plant responses to rising vapor pressure deficit. New Phytol. 2020:226(6):1550–1566. 10.1111/nph.16485 32064613

[kiaf337-B22] Hochberg U, Rockwell FE, Holbrook NM, Cochard H. Iso/anisohydry: a plant–environment interaction rather than a simple hydraulic trait. Trends Plant Sci. 2018:23(2):112–120. 10.1016/j.tplants.2017.11.002 29223922

[kiaf337-B23] Holbrook NM . Stem water storage. In: Gartner BL, editor. Plant stems. San Diego: Academic Press; 1995. p. 151–174. 10.1016/B978-0-12-276460-8.X5000-0

[kiaf337-B24] Hu Y, Sun Z, Zeng Y, Ouyang S, Chen L, Lei P, Deng X, Zhao Z, Fang X, Xiang W. Tree-level stomatal regulation is more closely related to xylem hydraulic traits than to leaf photosynthetic traits across diverse tree species. Agric For Meteorol. 2023:329:109291. 10.1016/j.agrformet.2022.109291

[kiaf337-B25] Javaux M, Carminati A. Soil hydraulics affect the degree of isohydricity. Plant Physiol. 2021:186(3):1378–1381. 10.1093/plphys/kiab154 33788931 PMC8260126

[kiaf337-B26] Jones HG . Monitoring plant and soil water status: established and novel methods revisited and their relevance to studies of drought tolerance. J Exp Bot. 2007:58(2):119–130. 10.1093/jxb/erl118 16980592

[kiaf337-B27] Jones MM, Turner NC. Osmotic adjustment in leaves of sorghum in response to water deficits. Plant Physiol. 1978:61(1):122–126. 10.1104/pp.61.1.122 16660224 PMC1091811

[kiaf337-B28] Klein T, Cohen S, Paudel I, Preisler Y, Rotenberg E, Yakir D. Diurnal dynamics of water transport, storage and hydraulic conductivity in pine trees under seasonal drought. iForest. 2016:9(5):710–719. 10.3832/ifor2046-009

[kiaf337-B29] Knipfer T, Bambach N, Hernandez MI, Bartlett MK, Sinclair G, Duong F, Kluepfel DA, McElrone AJ. Predicting stomatal closure and turgor loss in woody plants using predawn and midday water potential. Plant Physiol. 2020:184(2):881–894. 10.1104/pp.20.00500 32764130 PMC7536669

[kiaf337-B30] Knipfer T, Wilson N, Jorgensen-Bambach NE, McElrone AJ, Bartlett MK, Castellarin SD. Cessation of berry growth coincides with leaf complete stomatal closure at pre-veraison for grapevine (Vitis vinifera) subjected to progressive drought stress. Ann Bot. 2023:132(5):979–988. 10.1093/aob/mcad144 37742279 PMC10808015

[kiaf337-B31] Koehler T, Wankmüller FJ, Sadok W, Carminati A. Transpiration response to soil drying versus increasing vapor pressure deficit in crops: physical and physiological mechanisms and key plant traits. J Exp Bot. 2023:74(16):4789–4807. 10.1093/jxb/erad221 37354081 PMC10474596

[kiaf337-B32] Kramer PJ, Boyer JS. Water relations of plants and soils. San Diego: Academic Press; 1995.

[kiaf337-B33] Manandhar A, Pichaco J, McAdam SA. Abscisic acid increase correlates with the soil water threshold of transpiration decline during drought. Plant Cell Environ. 2024:47(12):5067–5075. 10.1111/pce.15087 39139139

[kiaf337-B34] Martínez-Vilalta J, Garcia-Forner N. Water potential regulation, stomatal behaviour and hydraulic transport under drought: deconstructing the iso/anisohydric concept. Plant Cell Environ. 2017:40(6):962–976. 10.1111/pce.12846 27739594

[kiaf337-B35] Martínez-Vilalta J, Poyatos R, Aguadé D, Retana J, Mencuccini M. A new look at water transport regulation in plants. New Phytol. 2014:204(1):105–115. 10.1111/nph.12912 24985503

[kiaf337-B36] McAdam SA, Brodribb TJ. The evolution of mechanisms driving the stomatal response to vapor pressure deficit. Plant Physiol. 2015:167(3):833–843. 10.1104/pp.114.252940 25637454 PMC4348763

[kiaf337-B37] McCutchan H, Shackel KA. Stem-water potential as a sensitive indicator of water stress in prune trees (Prunus domestica L. cv. French). J Am Soc Hortic Sci. 1992:117(4):607–611. 10.21273/JASHS.117.4.607

[kiaf337-B38] McDowell N, Pockman WT, Allen CD, Breshears DD, Cobb N, Kolb T, Plaut J, Sperry J, West A, Williams DG, et al Mechanisms of plant survival and mortality during drought: why do some plants survive while others succumb to drought? New Phytol. 2008:178(4):719–739. 10.1111/j.1469-8137.2008.02436.x 18422905

[kiaf337-B39] Mehlenbacher SA, Smith DC, McCluskey RL. ‘Yamhill’ hazelnut. HortScience. 2009:44(3):845–847. 10.21273/HORTSCI.44.3.845

[kiaf337-B40] Mehlenbacher SA, Smith DC, McCluskey RL. ‘Jefferson’ hazelnut. HortScience. 2011:46(4):662–664. 10.21273/HORTSCI.46.4.662

[kiaf337-B41] Mencuccini M, Anderegg WR, Binks O, Knipfer T, Konings AG, Novick K, Poyatos R, Martínez-Vilalta J. A new empirical framework to quantify the hydraulic effects of soil and atmospheric drivers on plant water status. Glob Chang Biol. 2024:30(3):e17222. 10.1111/gcb.17222 38450813

[kiaf337-B42] Mott KA, Peak D. Testing a vapour-phase model of stomatal responses to humidity. Plant Cell Environ. 2013:36(5):936–944. 10.1111/pce.12026 23072325

[kiaf337-B43] Muggeo VM . Segmented: an R package to fit regression models with broken-line relationships. R news. 2008:8(1):20–25. https://journal.r-project.org/articles/RN-2008-004/

[kiaf337-B44] Nobel PS . Physicochemical and environmental plant physiology. 4th ed. San Diego: Academic Press; 2009.

[kiaf337-B45] Novick KA, Ficklin DL, Baldocchi D, Davis KJ, Ghezzehei TA, Konings AG, MacBean N, Raoult N, Scott RL, Shi Y, et al Confronting the water potential information gap. Nat Geosci. 2022:15(3):158–164. 10.1038/s41561-022-00909-2 35300262 PMC8923290

[kiaf337-B46] Novick KA, Ficklin DL, Grossiord C, Konings AG, Martínez-Vilalta J, Sadok W, Trugman AT, Williams AP, Wright AJ, Abatzoglou JT, et al The impacts of rising vapour pressure deficit in natural and managed ecosystems. Plant Cell Environ. 2024:47(9):3561–3589. 10.1111/pce.14846 38348610

[kiaf337-B47] Pasqualotto G, Carraro V, Suarez Huerta E, Anfodillo T. Assessment of canopy conductance responses to vapor pressure deficit in eight hazelnut orchards across continents. Front Plant Sci. 2021:12:767916. 10.3389/fpls.2021.767916 34956266 PMC8692988

[kiaf337-B48] Passioura . Soil conditions and plant growth. Plant, Cell & Environ. 2002. 10.1046/j.0016-8025.2001.00802.x 11841672

[kiaf337-B49] Paternoster R, Brame R, Mazerolle P, Piquero A. Using the correct statistical test for the equality of regression coefficients. Criminology. 1998:36(4):859–866. 10.1111/j.1745-9125.1998.tb01268.x

[kiaf337-B50] Rodriguez-Dominguez CM, Brodribb TJ. Declining root water transport drives stomatal closure in olive under moderate water stress. New Phytol. 2020:225(1):126–134. 10.1111/nph.16177 31498457

[kiaf337-B51] Rodriguez-Dominguez CM, Buckley TN, Egea G, de Cires A, Hernandez-Santana V, Martorell S, Diaz-Espejo A. Most stomatal closure in woody species under moderate drought can be explained by stomatal responses to leaf turgor. Plant Cell Environ. 2016:39(9):2014–2026. 10.1111/pce.12774 27255698

[kiaf337-B52] Rodriguez-Dominguez CM, Hernandez-Santana V, Buckley TN, Fernández JE, Díaz-Espejo A. Sensitivity of olive leaf turgor to air vapour pressure deficit correlates with diurnal maximum stomatal conductance. Agric For Meteorol. 2019:272:156–165. 10.1016/j.agrformet.2019.04.006

[kiaf337-B53] Romero P, Botía P. Daily and seasonal patterns of leaf water relations and gas exchange of regulated deficit-irrigated almond trees under semiarid conditions. Environ Exp Bot. 2006:56(2):158–173. 10.1016/j.envexpbot.2005.01.012

[kiaf337-B54] Rosati A, Metcalf S, Buchner R, Fulton A, Lampinen B. Tree water status and gas exchange in walnut under drought, high temperature and vapour pressure deficit. J Hortic Sci Biotechnol. 2006:81(3):415–420. 10.1080/14620316.2006.11512082

[kiaf337-B55] Sack L, John GP, Buckley TN. ABA accumulation in dehydrating leaves is associated with decline in cell volume, not turgor pressure. Plant Physiol. 2018:176(1):489–495. 10.1104/pp.17.01097 29061906 PMC5761807

[kiaf337-B57] Scholander PF, Bradstreet ED, Hemmingsen EA, Hammel HT. Sap pressure in vascular plants: negative hydrostatic pressure can be measured in plants. Science. 1965:148(3668):339–346. 10.1126/science.148.3668.339 17832103

[kiaf337-B58] Shackel K . A plant-based approach to deficit irrigation in trees and vines. HortScience. 2011:46(2):173–177. 10.21273/HORTSCI.46.2.173

[kiaf337-B59] Shackel K, Moriana A, Marino G, Corell M, Pérez-López D, Martin-Palomo MJ, Caruso T, Marra FP, Agüero Alcaras LM, Milliron L, et al Establishing a reference baseline for midday stem water potential in olive and its use for plant-based irrigation management. Front Plant Sci. 2021:12:791711. 10.3389/fpls.2021.791711 34899813 PMC8663634

[kiaf337-B60] Shackel KA, Ahmadi H, Biasi W, Buchner R, Goldhamer D, Gurusinghe S, Hasey J, Kester D, Krueger B, Lampinen B, et al Plant water status as an index of irrigation need in deciduous fruit trees. HortTechnology. 1997: 7(1):23–29. 10.21273/HORTTECH.7.1.23

[kiaf337-B61] Sharma G, Brar GS, Knipfer T. Leaf water relations and osmotic adjustment of Canada western red spring wheat cultivars subjected to drought. Funct Plant Biol. 2023:50(12):1037–1046. 10.1071/FP23170 37814368

[kiaf337-B62] Soar CJ, Speirs J, Maffei SM, Penrose AB, McCarthy MG, Loveys BR. Grape vine varieties Shiraz and Grenache differ in their stomatal response to VPD: apparent links with ABA physiology and gene expression in leaf tissue. Aust J Grape Wine Res. 2006:12(1):2–12. 10.1111/j.1755-0238.2006.tb00038.x

[kiaf337-B63] Tardieu F, Simonneau T. Variability among species of stomatal control under fluctuating soil water status and evaporative demand: modelling isohydric and anisohydric behaviours. J Exp Bot. 1998:49(Special):419–432. 10.1093/jxb/49.Special_Issue.419

[kiaf337-B64] Tombesi S, Rechichi R, Lorusso R, Grisafi F. Relationship between shoot leaf area and nut set in hazelnut. X International Congress on Hazelnut 1379. 2023:297–302.

[kiaf337-B65] Turner NC, Long MJ. Errors arising from rapid water loss in the measurement of leaf water potential by the pressure chamber technique. Funct Plant Biol. 1980:7(5):527–537. 10.1071/PP9800527

[kiaf337-B66] Waring RH, Running SW. Sapwood water storage: its contribution to transpiration and effect upon water conductance through the stems of old-growth Douglas-fir. Plant Cell Environ. 1978:1(2):131–140. 10.1111/j.1365-3040.1978.tb00754.x

[kiaf337-B67] Wullschleger SD, Dixon MA, Oosterhuis DM. Field measurement of leaf water potential with a temperature-corrected in situ thermocouple psychrometer. Plant Cell Environ. 1988:11(3):199–203. 10.1111/j.1365-3040.1988.tb01137.x

