# Supplementary material for: Extension of the triphasic water potential curve: Accounting for air vapor pressure deficit under soil water stress
Source: Plant Physiol. 2025 Jul 31;199(1):kiaf337. doi: 10.1093/plphys/kiaf337 (PMC12415851; doi:10.1093/plphys/kiaf337)
Supplement: kiaf337_Supplementary_Data [file kiaf337_supplementary_data.pdf]

Supplemental Material

Table S1: Overview of environmental greenhouse conditions during experimental trials-1 (August 2022), -2 (September 2022), and -3 (July 2023).

Trial	T (°C)			RH (%)			VPD (kPa)		
	**mean**	**min**	**max**	**mean**	**min**	**max**	**mean**	**min**	**max**
1	24	19	28	68	57	79	0.96	0.47	1.47
2	22	18	28	56	44	70	1.20	0.7	1.93
3	29	24	32	45	41	61	2.24	1.2	2.65

Table S2. Parameters obtained from PV-curve analyses of hazelnut leaves. Data are mean ± standard error (n = 4 trees x 1-2 leaves per tree; total measurements = 6-7). For each parameter, *P*-values correspond to varietal comparison (π_o = osmotic pressure at full turgor; Ψ_{TLP} = leaf water potential at turgor loss point (TLP); a_f = apoplastic fraction of leaf water; '*' indicates the value is considering total leaf water storage, no '*' indicates the value is only considering symplastic water storage; RWC_{TLP} = relative water content at TLP, ε = bulk modulus of elasticity at full turgor, SWC = saturation water content (mass of water per dry mass of leaf), C = relative capacitance at TLP and full turgor (FT), $C_{FT, absolute}$ = absolute capacitance at full turgor per leaf area.)

Variety	π_o (MPa)	Ψ_{TLP} (MPa)	a_f (%)	RWC^*_{TLP} (%)
**Jefferson**	1.13 ± 0.02	-1.76 ± 0.06	50.7 ± 3.71	83.0 ± 0.97
**Yamhill**	1.41 ± 0.07	-2.06 ± 0.08	33.42 ± 4.07	79.24 ± 1.34
	P = 0.003	*P* = 0.007	*P* = 0.005	*P* = 0.124
**Variety**	**RWC_{TLP} (%)**	**ε* (MPa)**	**ε (MPa)**	**Leaf Area (cm^2)**
**Jefferson**	64.8 ± 2.49	6.23 ± 0.54	3.04 ± 0.30	76.5 ± 7.81
**Yamhill**	68.6 ± 1.78	6.60 ± 0.63	4.34 ± 0.41	51.0 ± 5.82
	P = 0.024	*P* = 0.332	*P* = 0.014	*P* = 0.013
**Variety**	**SWC**	**C^*_{FT} (MPa^{-1})**	**C^*_{TLP} (MPa^{-1})**	
**Jefferson**	2.35 ± 0.19	0.10 ± 0.01	0.11 ± 0.02	
**Yamhill**	1.56 ± 0.03	0.11 ± 0.01	0.13 ± 0.02	
	P = 0.004	*P* = 0.316	*P* = 0.183	
**Variety**	**C_{FT} (MPa^{-1})**	**C_{TLP} (MPa^{-1})**	**$C_{FT, absolute}$ (mol m^{-2} MPa^{-1})**	
**Jefferson**	0.21 ± 0.01	0.21 ± 0.02	0.69 ± 0.06	
**Yamhill**	0.16 ± 0.01	0.19 ± 0.02	0.69 ± 0.08	
	P = 0.010	*P* = 0.285	*P* = 0.483	

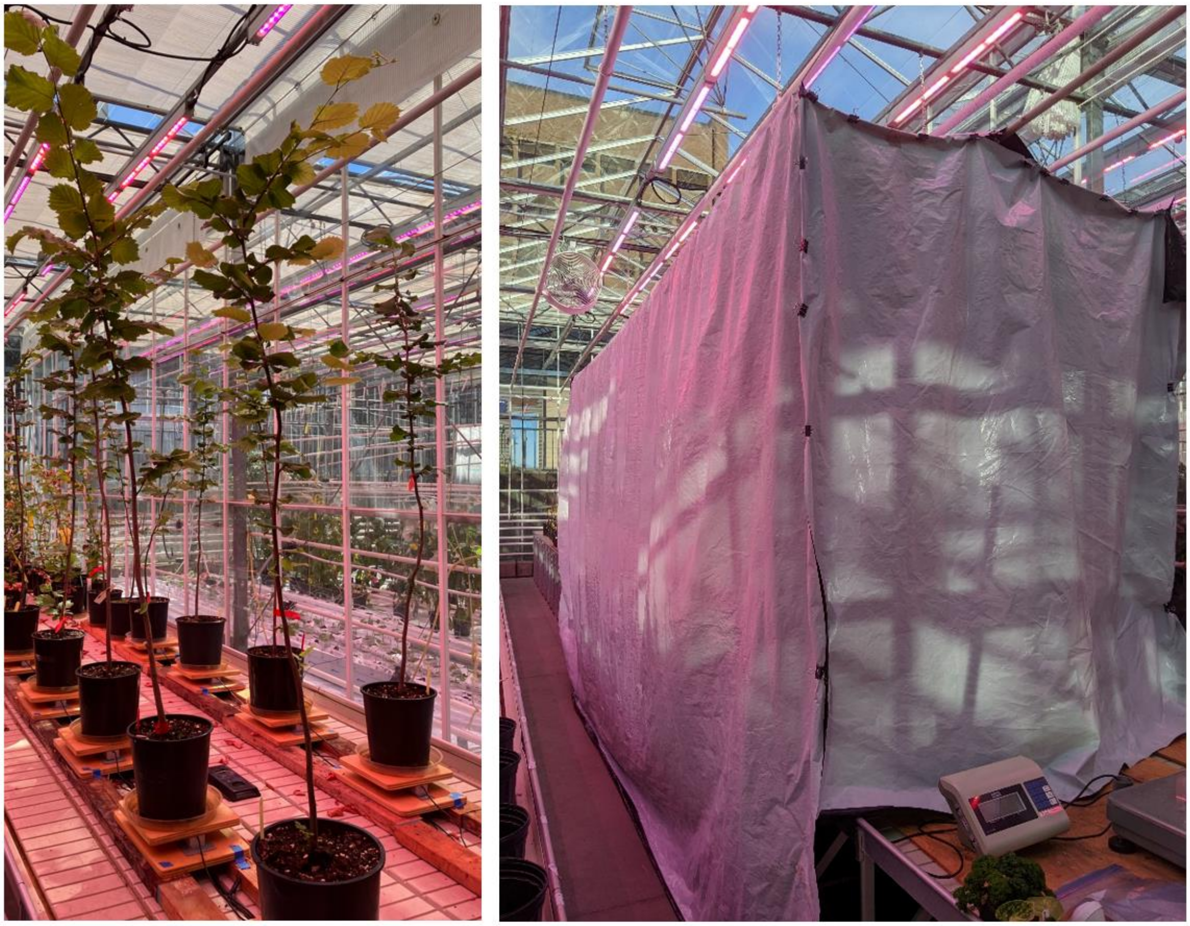

Figure S1. Representative image of hazelnut trees placed on mini-weighing lysimeters taken August 10, 2022 (left hand image). A plastic cover was pulled over the bench to exclude light exposure and minimize nighttime transpiration prior to predawn water potential measurements (right hand image).

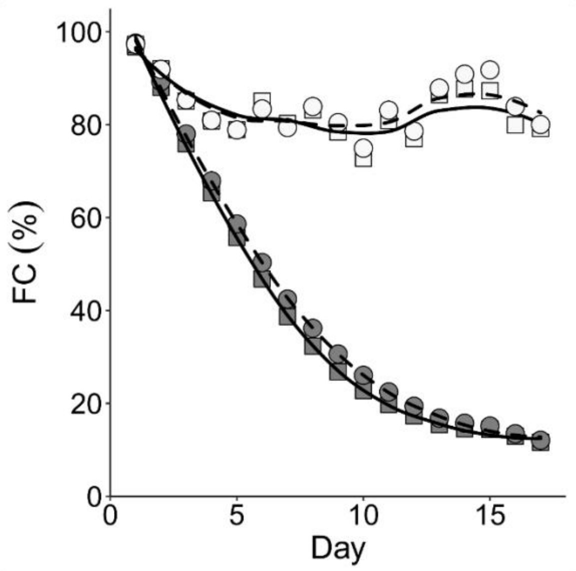

Figure S2. Representative time course of changes in percentage of field capacity (FC) when pooled for experimental trials-1 and -2 ('Jefferson' = squares symbols, solid line; 'Yamhill' = circle symbols and dashed line). Each symbol is the mean of n = 4-8 trees (white color = fully-irrigated trees; grey color = dry-down trees). Lines are locally estimated scatterplot smoothing lines to help with visualizing the change in FC over time.

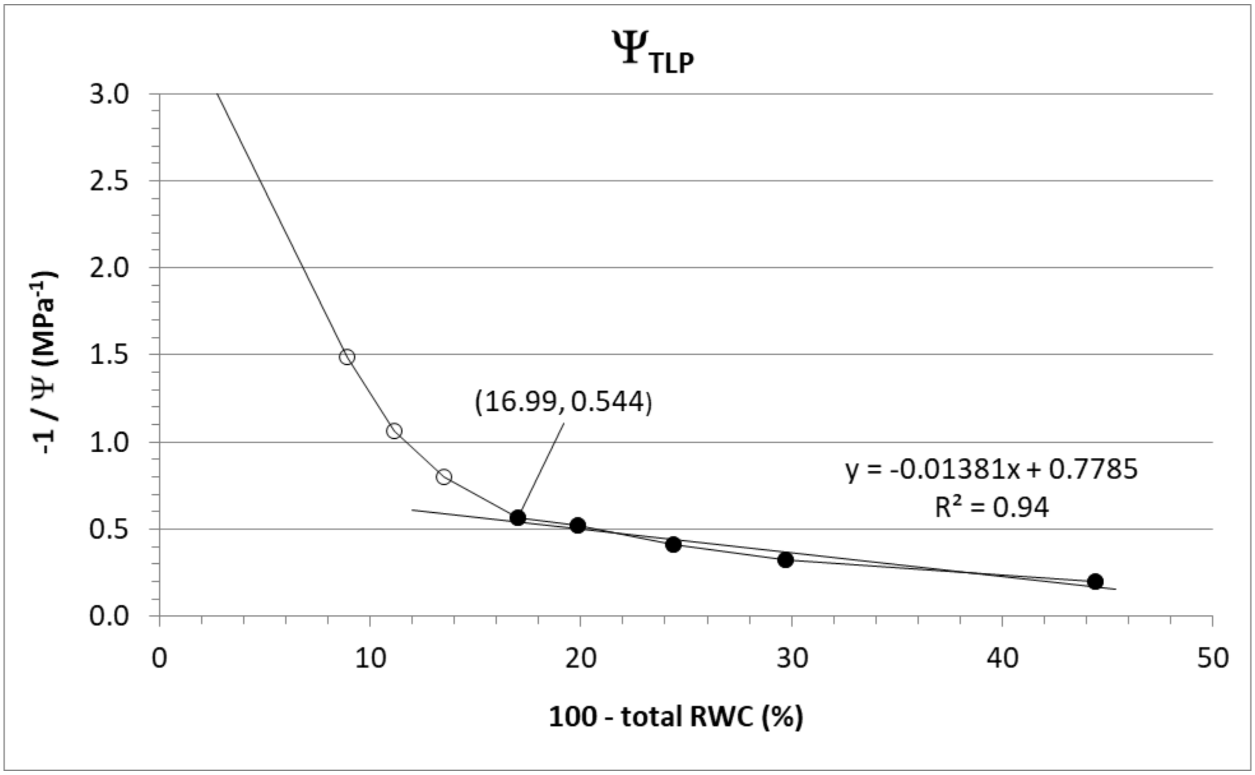

Figure S3: Representative PV-curve to derive TLP_{PV} ('Yamhill'). Each symbol is a single measurement and the entire figure represents one leaf. The curved line is for visualization only; the straight line is a regression line used to identify the change in the relationship between a reduction of leaf water content and the associated change in the negative inverse of the leaf water potential using the R^2. The TLP_{PV} for this leaf was identified as the negative inverse of the expected y-value of the model for the identified point (i.e., TLP_{PV} = -1 / 0.544 = 1.838 MPa).
